# Neoadjuvant pyrotinib for HER2 (IHC 2+/FISH+)/HR+ breast cancer with early non-response: a case report and literature review

**DOI:** 10.3389/fmed.2026.1805199

**Published:** 2026-07-24

**Authors:** Jiani Liu, Xinyu Peng, Xuhui Huang, Jirui Sun, Dan Geng

**Affiliations:** 1 Department of Breast Surgery, Baoding No. 1 Central Hospital, Baoding, Hebei, China; 2 Department of Gastrointestinal Surgery, Affiliated Hospital of Hebei University, Baoding, Hebei, China; 3 Department of Pathology, Baoding First Central Hospital, Baoding, Hebei, China

**Keywords:** breast cancer, HER2-positive, neoadjuvant, non-response, pyrotinib

## Abstract

Neoadjuvant therapy (NAT) has been widely adopted in the multimodal management of HER2-positive breast cancer. Trastuzumab and pertuzumab (HP) combined with chemotherapy is the recommended neoadjuvant regimen for patients with HER2-positive locally advanced breast cancer. Achieving total pathological complete response (tpCR) after NAT is a strong predictor of long-term survival in patients with early-stage or locally advanced breast cancer. Nevertheless, approximately 40% of patients fail to achieve tpCR with this standard approach. Early identification of limited or non-responders and switching to a pyrotinib-containing regimen to improve tpCR currently lack a unified clinical standard. Here, we report a case of HER2 (IHC 2+/FISH+)/hormone receptor (HR)+ breast cancer that was assessed as limited early response after two cycles of standard therapy. The regimen was modified to trastuzumab plus pyrotinib plus nab-paclitaxel, resulting in total pathological complete response (tpCR) with good tolerability; only mild, controllable diarrhea occurred. This response-adapted strategy, based on early response assessment by chest CT, warrants further validation.

## 1 Introduction

HER2-positive breast cancer accounts for approximately 15%–20% of all breast cancers worldwide (1), and has been reported to be 22.5% in Chinese cohorts (2). HER2-positive disease is biologically aggressive, however, the efficacy and prognosis have improved substantially over the past decades with the rapid evolution of anti-HER2 agents. NAT has been widely implemented for HER2-positive breast cancer. Evidence indicates that tpCR after NAT in early-stage or locally advanced breast cancer is a strong predictor of long-term survival. The NeoSphere (3) and PEONY (4) trials established trastuzumab plus pertuzumab (HP) as the backbone of neoadjuvant anti-HER2 therapy. Studies including TRAIN-2 and others (5–8) have shown that HP combined with chemotherapy can increase tpCR rates to approximately 60%. Nonetheless, breast cancer is highly heterogeneous. HER2-positive breast cancer comprises distinct subtypes, such as HR-positive versus HR-negative disease, and HER2 IHC 3+ versus HER2 IHC 2+/FISH+ disease, which respond differently to anti-HER2 therapy. Krystel-Whittemore et al. reported significantly higher tpCR rates in IHC 3+ tumors than in IHC < 3+/FISH+ tumors (67.0% vs. 17.0%, *P* < 0.0001) (9), and similar findings have been observed in retrospective analyses (10, 11). In NeoSphere (3), the tpCR rate with THP (docetaxel, trastuzumab, and pertuzumab) was 63.2% in HR-negative tumors versus 26% in HR-positive tumors; comparable patterns have been reported elsewhere (6–8). In a real-world study by Xiang et al., the pCR rate after HP-based neoadjuvant chemotherapy was 67.4% in HER2 IHC 3+/HR- tumors but only 17.1% in HER2 (IHC 2+/FISH+)/HR+ tumors (12). However, current domestic and international guidelines do not differentiate neoadjuvant strategies across these HER2-positive subtypes. As outcomes continue to improve, there is an increasing need to refine precision and individualized treatment to further enhance efficacy.

Pyrotinib (Py) is a domestically developed pan-ErbB tyrosine kinase inhibitor that irreversibly binds to the intracellular tyrosine kinase domains of HER1, HER2, and HER4 through covalent interaction with the ATP-binding site. It broadly prevents formation of HER family homo-/heterodimers, inhibits substrate phosphorylation, and blocks downstream signaling. The NeoPaTHer study evaluated early response using breast MRI after two cycles of TCbH (docetaxel, carboplatin, and trastuzumab). Responders were assigned to group A; non-responders could either continue the original regimen (group B) or receive additional pyrotinib (group C). The tpCR rates were 30.6% in group A, 15.4% in group B, and 29.3% in group C. Multivariable logistic regression suggested comparable odds of achieving tpCR in groups A and C (13), implying that pyrotinib nearly doubled the tpCR rate among early non-responders. In the current era of HP-based neoadjuvant therapy, whether early identification of non or limited-responders and switching to a combined large- and small-molecule regimen (HPy) can improve tpCR and prognosis warrants further study.

This case report describes a patient with locally advanced HER2 (IHC 2+/FISH+)/HR+ breast cancer who was assessed as limited early response after two cycles of standard therapy. The regimen was switched to trastuzumab plus pyrotinib plus chemotherapy, resulting in tpCR. Please note that in this study, the definition of tpCR is as: no invasive carcinoma remaining in the breast primary lesion, with ductal carcinoma *in situ* allowed, and negative axillary lymph nodes (ypT0/is ypN0). This corresponds to either a Miller-Payne grade 5 in the primary lesion with negative lymph nodes, or a Residual Cancer Burden (RCB) class 0.

## 2 Case report

**FIGURE 1:**
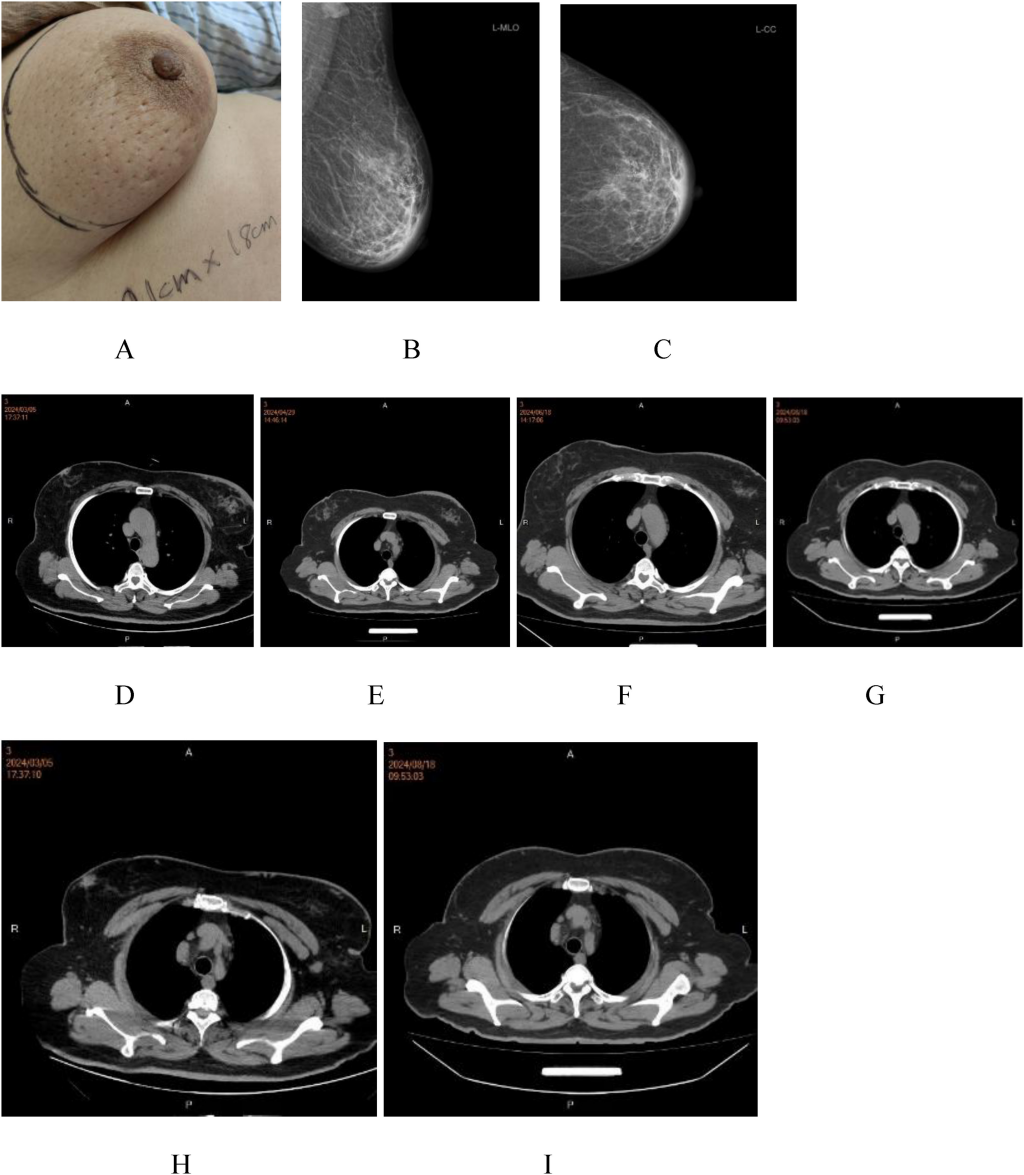
Appearance of the breasts, mammography at initial diagnosis, and computed tomography (CT) images. **(A)** Photograph of the breasts at initial diagnosis. **(B)** Mammography at initial diagnosis (mediolateral oblique view, MLO). **(C)** Mammography at initial diagnosis (craniocaudal view, CC). Mammography revealed central breast skin edema and thickening, with segmental microcalcifications spanning approximately 5.0 cm × 3.5 cm. **(D)** CT mediastinal window at initial diagnosis (5 March 2024) showed a hyperdense shadow in the left breast measuring approximately 60 mm × 25 mm, with skin edema and thickening. **(E)** After two cycles of the THP regimen (29 April 2024), CT showed the lesion measuring approximately 53.4 mm × 24 mm, with persistent skin edema and thickening. **(F)** After two cycles of the THPy regimen (18 June 2024), CT revealed a hyperdense shadow measuring approximately 33 mm × 19 mm, with resolution of skin edema. **(G)** After four cycles of the THPy regimen (18 August 2024), CT showed a slightly hyperdense shadow measuring approximately 23 mm × 12 mm, with no evidence of skin edema. **(H)** CT mediastinal window at initial diagnosis showed enlarged axillary lymph nodes. **(I)** CT mediastinal window on 18 August 2024 showed no abnormal lymph node shadow.

**FIGURE 2:**
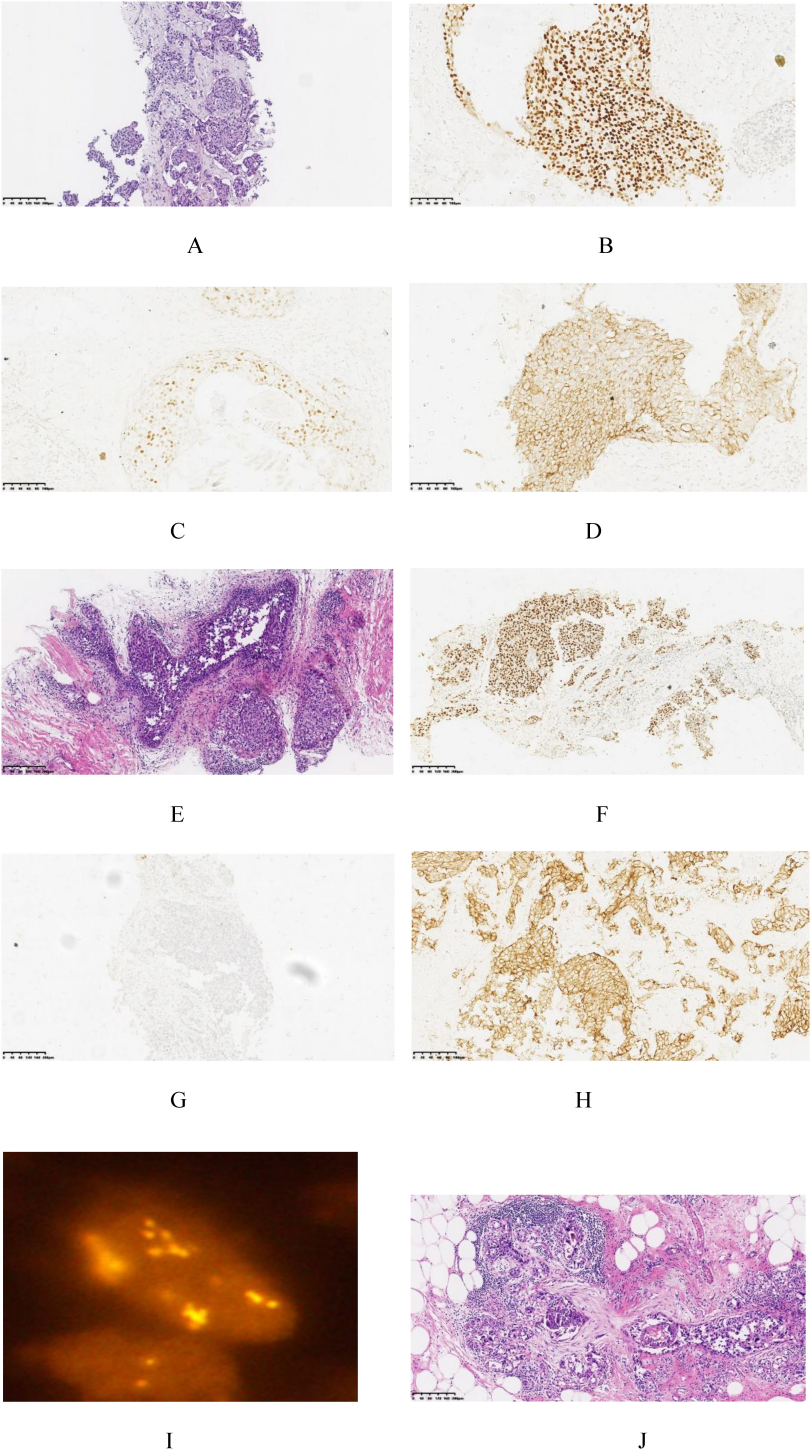
Pathology and immunohistochemistry images of the patient. **(A)** Core needle biopsy of the breast mass (March 2024, H&E staining, ×100) showed invasive ductal carcinoma. **(B–D)** Immunohistochemical staining of the breast mass (×200) revealed estrogen receptor (ER) (+++), progesterone receptor (PR) (+), and HER2 (2+), respectively. **(E)** Core needle biopsy of the left axillary lymph node (March 2024, H&E staining, ×100) showed metastatic carcinoma. **(F–H)** Immunohistochemical staining of the left axillary lymph node (×100 for ER and PR; ×200 for HER2) revealed ER (90%), PR (–), and HER2 (2+), respectively. **(I)** Fluorescence *in situ* hybridization (FISH) confirmed HER2 gene amplification. **(J)** Postoperative histopathology revealed tumor atrophy and degeneration, with only residual ductal carcinoma *in situ* (DCIS) remaining.

A 55-year-old Chinese woman presented to our hospital in March 2024 with a 15-day history of redness, swelling, and thickening of the left breast skin accompanied by a palpable mass in the upper outer quadrant. On examination, peau d’orange changes were observed over an area of 21.0 cm × 18.0 cm in the central breast (Figure 1A), with a palpable 4.0 cm × 3.5 cm mass in the upper outer quadrant. A palpable enlarged left axillary lymph node measuring 2.0 cm × 2.0 cm was noted. Medical history included grade 3 hypertension and type 2 diabetes mellitus, both well-controlled. Core needle biopsy of the breast mass showed invasive ductal carcinoma. Immunohistochemistry (IHC) revealed estrogen receptor (ER) (+++), progesterone receptor (PR) (+), HER2 (2+), and Ki-67 (20%). Fluorescence *in situ* hybridization (FISH) confirmed HER2 gene amplification. Core needle biopsy of the left axillary lymph node demonstrated metastatic carcinoma, with ER (90%), PR (−), HER2 (2+), and Ki-67 (20%+) (Figure 2). After comprehensive evaluation, the clinical stage was determined as T4N1M0, stage IIIB.

**FIGURE 3:**
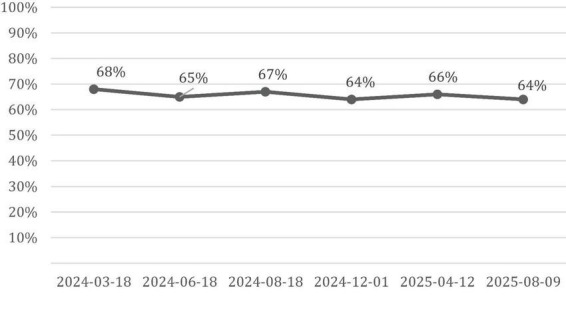
Time points and corresponding values of left ventricular ejection fraction (LVEF).

**FIGURE 4:**
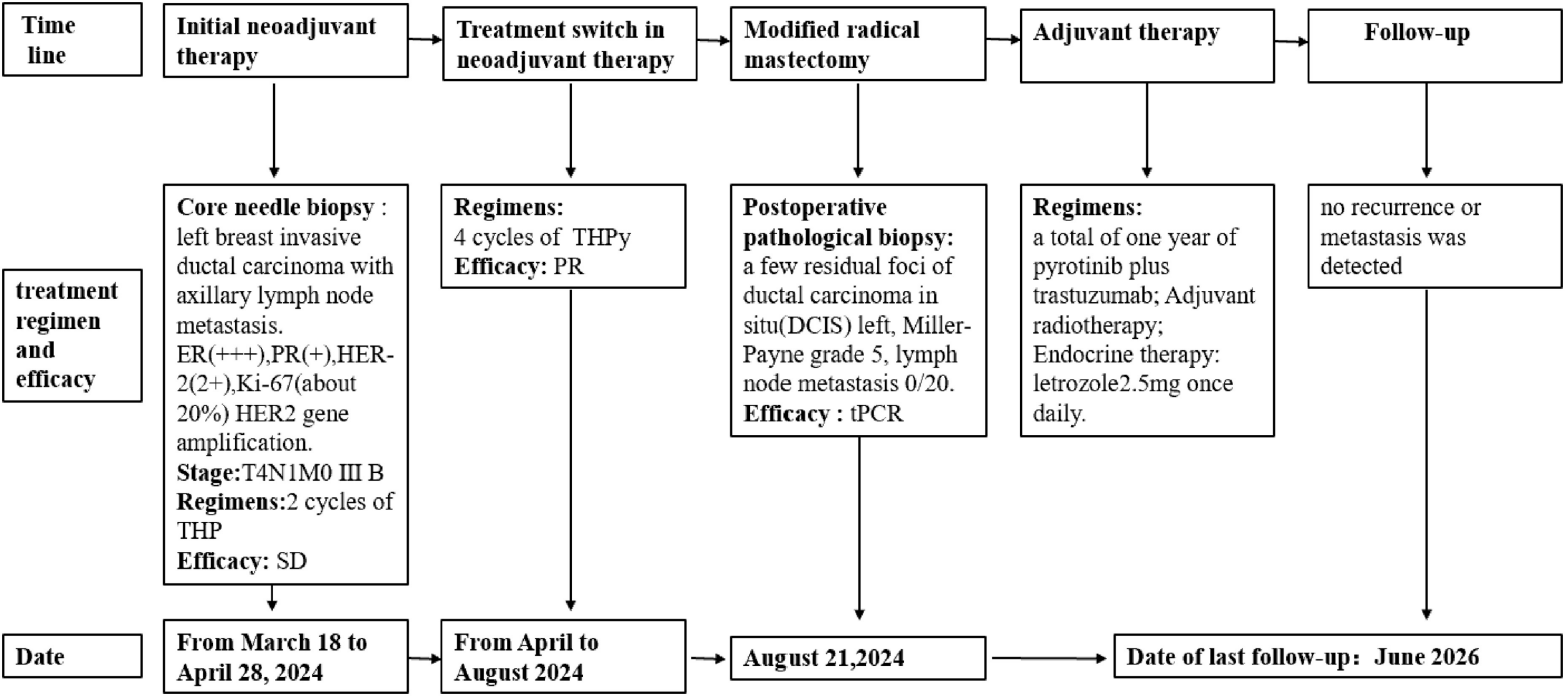
Timeline of this patient. IHC, immunohistochemistry; ER, estrogen receptor; PR, progesterone receptor; HER2, human epidermal growth factor receptor 2; FISH, fluorescence *in situ* hybridization; SD, stable disease; PR, partial response; tpCR, total pathological complete response.

The patient was 158 cm in height and weighed 80 kg, with a body surface area (BSA) of 1.83 m^2^. From 18 March to 28 April 2024, the patient received two cycles of nab-paclitaxel, trastuzumab, and pertuzumab (THP). Doses were as follows: nab-paclitaxel 450 mg (246 mg/m^2^; relative dose intensity 94.6%), trastuzumab 640 mg in cycle 1 followed by 480 mg in subsequent cycles; pertuzumab 840 mg in cycle 1 followed by 420 mg in subsequent cycles; administered every 3 weeks. On 29 April 2024, response assessment based on physical examination and computed tomography (CT) according to RECIST 1.1 indicated stable disease (SD). CT revealed that the longest diameter of the tumor decreased from 60.0 to 53.4 mm after treatment, representing an 11% reduction, while no improvement was observed in skin erythema, edema, thickening, or peau d’orange appearance. Although continuing the current THP regimen with reassessment after additional cycles might have been a reasonable alternative, the regimen was switched to THPy given the need for rapid symptom relief. The patient received four cycles of treatment with nab-paclitaxel 450 mg every 3 weeks, subcutaneous trastuzumab 600 mg every 3 weeks, and oral pyrotinib 320 mg once daily. The switch from intravenous to subcutaneous trastuzumab was performed to enable completion of 1 year of adjuvant targeted therapy in the outpatient setting without hospitalization. The patient responded well. With loperamide prophylaxis, only occasional mild diarrhea occurred. CT showed that the longest diameter of the lesion decreased from 53.4 to 33.0 mm after two cycles of the THPy regimen, representing a 38.2% reduction. Complete resolution of skin erythema and edema was observed, along with a marked decrease in the extent of peau d’orange. After four cycles, neither the left breast tumor nor the left axillary nodes were palpable; peau d’orange changes and swelling disappeared. Ultrasound showed a 1.4 cm heterogeneous hypoechoic area. CT demonstrated a slightly hyperdense region of approximately 2.3 cm × 1.2 cm at the original tumor site, with a 56.9% reduction in size after four cycles of THPy and resolution of previously noted axillary lymphadenopathy and skin thickening. The overall response was partial response (PR) (Figure 1). Given the marked shrinkage, CT was also used to assess the relationship between axillary nodes and vessels and to evaluate surgical feasibility. On 21 August 2024, the patient underwent modified radical mastectomy. Postoperative pathology (left breast and axillary tissues) showed a few residual foci of ductal carcinoma *in situ* (DCIS) in the breast, with a maximum microscopic diameter of 1.3 cm, Miller–Payne grade 5, and IHC findings of ER (+++), PR (+), HER2 (2+), and Ki-67 (20%) (Figure 2). No lymph node metastasis was identified (axillary 0/15; interpectoral 0/1; apical 0/4). Given that tpCR was achieved, the patient received adjuvant pyrotinib and trastuzumab postoperatively to complete a 1-year treatment course. Throughout treatment, only mild diarrhea and peripheral numbness were noted; these symptoms were effectively controlled with supportive care, and no serious adverse events occurred. Left ventricular ejection fraction (LVEF) was assessed regularly throughout the treatment period, with no significant alterations observed (Figure 3). The patient also received postoperative radiotherapy and endocrine therapy with letrozole 2.5 mg once daily. At 22 months after surgery, no recurrence or metastasis was detected (timeline shown in Figure 4). All procedures complied with institutional/national ethics standards and the Declaration of Helsinki (2013 revision). Written informed consent was obtained for publication of this case report and accompanying images.

## 3 Discussion

*In vitro* studies have shown that pyrotinib, alone or in combination with trastuzumab, inhibits proliferation of trastuzumab-resistant HER2-positive breast cancer cells; *in vivo* studies similarly demonstrated suppression of trastuzumab-resistant xenograft growth (14). In contrast, pertuzumab alone or combined with trastuzumab failed to reverse resistance in trastuzumab-resistant models (14–16). Multiple molecular mechanisms may underlie low sensitivity, including proteolytic cleavage of the HER2 extracellular (N-terminal) domain generating p95HER2; upregulation of mucin 4 (MUC4), which hinders trastuzumab recognition and binding to key HER2 epitopes; and activation of bypass signaling pathways downstream of HER2, such as IGF-1R upregulation activating PI3K/AKT and RAS pathways, thereby diminishing responsiveness. Pyrotinib inhibits activation of HER2 downstream PI3K/AKT and RAS/RAF/MEK/ERK pathways intracellularly (14). The combination of trastuzumab and pyrotinib—large and small-molecule agents—may synergistically block HER2 signaling extracellularly and intracellularly, resulting in improved benefit. Therefore, based on preclinical evidence, patients with trastuzumab low sensitivity derive greater benefit from pyrotinib than from pertuzumab.

In the first-line metastatic setting, the CLEOPATRA trial reported a median progression-free survival (mPFS) of 16.9 months for patients receiving THP who had previously been treated with trastuzumab in the (neo)adjuvant setting, compared with 21.6 months in trastuzumab-naïve patients (17). In the PHILA trial, mPFS reached 24.3 months in the THPy group; subgroup analyses suggested that mPFS was not reached (NR) among trastuzumab-pretreated patients receiving pyrotinib, while mPFS was 21.9 months among trastuzumab-naïve patients in the pyrotinib group (18). The PANDORA trial of pyrotinib plus docetaxel yielded similar findings: trastuzumab-pretreated patients had longer mPFS than trastuzumab-naïve patients (20.8 vs. 14.8 months) (19). Admittedly, extrapolation from metastatic endpoints to the neoadjuvant setting is not feasible. But these data indirectly suggest that the combination of pyrotinib and trastuzumab may, to some extent, be more effective than pertuzumab in overcoming resistance to anti-HER2 therapy. However, metastatic disease remains incurable; thus, incorporating pyrotinib and trastuzumab earlier in selected populations in the (neo)adjuvant setting to improve tpCR or invasive disease-free survival (iDFS) is worth further investigation.

The NeoPaTHer study showed that adding pyrotinib nearly doubled the tpCR rate among TCbH early non-responders (13), suggesting that pyrotinib may partially overcome suboptimal response to anti-HER2 therapy during neoadjuvant treatment. Beyond adjusting neoadjuvant decisions based on early clinical response, precisely identifying sensitive populations and developing accurate biomarkers to predict pyrotinib efficacy are key research priorities. Unfortunately, the NeoPaTHer study did not include pertuzumab in the initial treatment regimen and therefore cannot directly demonstrate that switching to a pyrotinib-containing regimen is superior to pertuzumab. Further clinical studies are needed to validate this.

In the PHEDRA trial, in the HR-positive subgroup, tpCR was more than doubled with TH+Py versus TH alone (29.9% vs. 12.2%) (20); by contrast, in NeoSphere, the improvement in tpCR with TH+P over TH in HR-positive tumors was modest (26% vs. 20%) (3). Although cross-trial comparisons should be interpreted with caution, these data indirectly support the hypothesis that pyrotinib-containing regimens may be more effective than pertuzumab-containing regimens in improving outcomes in HR+ breast cancer. In the Panphila study, after neoadjuvant TCbHPy, tpCR was higher in HER2 IHC 3+ tumors than in HER2 IHC 2+/FISH(+) tumors (60.7% vs. 30.8%) (21). Real-world data similarly showed a pCR rate of 67.4% in HER2 IHC 3+/HR− tumors after HP but only 17.1% in HER2 (IHC 2+/FISH+)/HR+ tumors (12). Other retrospective studies have reported consistent patterns, and HER2 IHC 3+ is an independent predictor of higher tpCR (22). Although head-to-head trials are lacking, available evidence suggests pyrotinib may increase tpCR rates in IHC 2+/FISH+ tumors. Therefore, a regimen of pyrotinib plus trastuzumab plus chemotherapy may improve pCR in patients with HER2 (IHC 2+/FISH+)/HR+ disease with poor response, and subtype-guided precision strategies may further enhance outcomes and prognosis in HER2-positive breast cancer. The literature provides only indirect support for this specific substitution; therefore, this approach should be framed as hypothesis-generating.

In this case, the discontinuation of pertuzumab was based not only on concerns regarding cumulative adverse events associated with multidrug regimens, but also on cost-effectiveness considerations, as well as the relatively robust clinical evidence and theoretical rationale supporting the THPy regimen. Nonetheless, the potential for enhanced efficacy with the addition of pyrotinib to an HP-based backbone remains clinically relevant and warrants further investigation.

Contrast-enhanced MRI (CE-MRI) is generally considered the most accurate imaging modality for assessing breast lesions. In clinical practice in China, however, CT is more economical and convenient. Although CT may not identify microcalcifications, this limitation does not preclude its utility in response assessment (23). Furthermore, CT enables evaluation of the relationship between axillary lymph nodes and adjacent vessels, which supports preoperative planning. Accordingly, chest CT is commonly used to evaluate treatment response in breast cancer in many primary or resource-limited hospitals in China.

Regarding adverse events, diarrhea is the most common toxicity associated with pyrotinib-containing regimens (incidence 88%–100%), and grade ≥ 3 diarrhea occurs in 40%–45%, representing the main cause of dose reduction, treatment delays, or discontinuation. In PHILA study, 26.0% of patients required pyrotinib dose reduction (18). In Panphila, after 12 patients were enrolled in the single-arm TCbHPy cohort, the pyrotinib dose was reduced to 320 mg in subsequent enrollments due to diarrhea-related tolerability concerns. (21). Despite pyrotinib dose reduction, a pCR rate of 55.1% was achieved in this study. In this case, to reduce perceived adverse events and improve patient adherence, pyrotinib was initiated at a dose of 320 mg with strict primary prophylaxis (loperamide) initiated upon grade 1 diarrhea. The patient experienced only occasional mild diarrhea. As nab-paclitaxel monotherapy was used for chemotherapy, nausea/vomiting and myelosuppression were not observed; only mild peripheral neuropathy occurred.

In this case, the patient initially presented with unresectable stage IIIB HER2-positive locally advanced breast cancer (T4N1M0), for which neoadjuvant therapy is appropriate. However, the molecular subtype was HER2 (IHC 2+/FISH+)/HR+, for which numerous studies have shown suboptimal response to standard neoadjuvant therapy; nevertheless, current guidelines and routine clinical practice do not differentiate treatment by this subtype. After guideline-recommended THP was administered at our institution, the response after two cycles was poor. We promptly switched to THPy based on early response evaluation. To the best of our knowledge, this is the first case report of HER2 (IHC 2+/FISH+)/HR-positive locally advanced breast cancer in which the neoadjuvant regimen was switched to a pyrotinib-containing strategy based on limited early response. With nab-paclitaxel monochemotherapy, the patient achieved tpCR and demonstrated favorable safety. This case suggests that, for this specific population, response-guided precision evaluation and pyrotinib combined with trastuzumab and nab-paclitaxel may provide substantial efficacy with a lower risk of adverse events. However, further studies in larger cohorts are required to confirm efficacy and response patterns.

Limitations in this case report: Firstly, precise definitions of “limited response” could not be established in a single case, and their clinical utility warrants validation in larger cohorts; Secondly, RCB assessment was not routinely incorporated into pathology reports at our institution at the time of diagnosis; Thirdly, although one year of adjuvant HPy therapy is widely adopted as a treatment duration in clinical trials, long-term follow-up data from clinical studies remain limited.

## 4 Conclusion

For patients with limited early response to HP-based neoadjuvant therapy, no standardized regimen for treatment switching is currently available. In this case, switching from pertuzumab to pyrotinib, while maintaining trastuzumab and chemotherapy demonstrated feasibility in a patient with HER2 (IHC 2+/FISH+)/HR+ breast cancer who showed a suboptimal early response. However, as this finding is derived from a single case, further well-designed clinical trials are warranted to evaluate the effectiveness of such response-adapted treatment strategies.

## Data Availability

The original contributions presented in this study are included in this article/supplementary material, further inquiries can be directed to the corresponding author.
